# Assessing intra-rater reliability of peripheral quantitative computed tomography in knee joint bone evaluation on individuals with and without obesity: A GRRAS study

**DOI:** 10.1016/j.ostima.2026.100393

**Published:** 2026-03-07

**Authors:** Reece Blay, Christopher Wichman, Yvonne M. Golightly, Laura Bilek

**Affiliations:** a College of Allied Health Professions, University of Nebraska Medical Center, Omaha, NE, USA; b College of Public Health, University of Nebraska Medical Center, Omaha, NE, USA

**Keywords:** Knee Osteoarthritis, Obesity, Imaging

## Abstract

**Objective:**

Common imaging approaches for knee osteoarthritis (KOA) do not reliably assess early bone changes. Obesity is a major KOA risk factor and can complicate imaging due to positioning and image quality. This pilot study primarily aimed to assess the intra-rater reliability of peripheral quantitative computed tomography (pQCT) for evaluating bone at the distal femur and proximal tibia, with a secondary objective of comparing reliability metrics between individuals with and without obesity.

**Design:**

52 participants (81% female, mean age 50 [range 23-79] years) were matched by body mass index (BMI) (obese: BMI ≥ 30 kg/m^2^, non-obese: BMI < 30 kg/m^2^), within ± 5 years of age, sex, and >5 kg/m^2^ BMI difference. A single technician acquired two independent pQCT scans (Stratec XCT 3000) of the right tibiofemoral joint during one visit. Reliability was assessed using coefficient of variation root mean squared (CV_rms_), reliability coefficient (R), and Bland-Altman analyses for repeated bone density (total, trabecular, cortical) and cross-sectional area (CSA) measures overall and by obesity group.

**Results:**

pQCT demonstrated no systematic bias overall, with no substantial differences in reliability between obesity groups. Density measures showed excellent precision at all tibial regions and the medial femoral condyle. Tibial CSA measures showed no systematic bias but low precision. CSA measures at the medial femoral condyle were unreliable, and all measures at the lateral femoral condyle were unreliable due to inconsistent image capture.

**Conclusion:**

pQCT is a potential tool to reliably detect early KOA-related bone changes, with no substantial differences in reliability between individuals with and without obesity.

## Introduction

Knee osteoarthritis (KOA), a leading cause of disability in the U.S., is marked by joint pain, stiffness, and reduced mobility, often resulting in fatigue, loss of function, work limitations, and diminished quality of life [1,2]. Disability and economic costs of KOA are expected to increase as obesity prevalence rises since persons with obesity have nearly seven times the risk of developing KOA compared to those without obesity [3]. While there are therapies to decrease pain and improve function for persons with KOA, there are no therapies for halting the disease once initiated. Therefore, prevention of KOA is key to addressing the disease. This is difficult as the mechanisms of the disease are not well elucidated.

Bone changes occur throughout the initiation and progression of KOA [[4], [5], [6]]. Early pathological changes include thinning of the subchondral bone resulting in a decreased bone mineral density of the area [7]. As the process progresses, there is a contrasting increase in density of the subchondral bone in both the tibia and femur [8,9]. Changes in bone density are associated with cartilage loss and KOA progression [6,8,10]. While these changes have been observed in animal models and post-injury in humans within a short timeframe (following individuals for 2 years or less) [[11], [12], [13]], there is a lack of evidence about the temporal progression of bone changes in the development of KOA in humans. This is due in part to a lack of sensitive and convenient imaging tools to monitor bone changes before and during the initial phases of KOA.

KOA is diagnosed through physical examination and patient-reported signs and symptoms [14]. The primary method for tracking KOA structural progression is through radiographic imaging using the Kellgren-Lawrence grading scale [15]. This scale categorizes KOA stages based on joint space narrowing and osteophyte formation [15]. However, radiographs may not detect early disease changes in bone since joint space narrowing and osteophyte formation occur after the bones have already become sclerotic [16]. This limitation suggests that radiography may miss important early changes in the disease process, which is a crucial window for potential prevention. Additionally, radiographs lack detailed information on bone density and structure (trabecular vs cortical bone). Dual-energy X-ray absorptiometry (DXA) can assess bone density but shares similar limitations to radiographs since it cannot assess internal bone features. Magnetic Resonance Imaging (MRI) offers detailed information about soft tissues but is less reliable for bone assessment [17]. Quantitative Computed Tomography (QCT) can measure bone density and provides detailed structural data, but it's not widely standardized for knee assessment, exposes patients to high doses of radiation, and is expensive and time-consuming [18,19]. Therefore, an alternative tool is needed to evaluate the early bone features of KOA.

Obtaining images from individuals with obesity also presents further challenges [[20], [21], [22]]. The presence of excess adipose tissue can affect image quality by absorbing and scattering X-rays [20,22]. This interference reduces image clarity and detail [[20], [21], [22]]. Individuals with obesity often need a higher radiation dose during CT scans to produce satisfactory images, raising concerns about radiation exposure and prolonged imaging sessions [[20], [21], [22]]. Moreover, obesity complicates imaging due to difficulties in positioning individuals and identifying landmarks for precise imaging [20]. The combination of these factors makes it more challenging to obtain reliable images of individuals with obesity. Peripheral quantitative computed tomography (pQCT) has emerged as a valuable tool for assessing bone density and structure of the limbs, offering a compelling alternative to traditional imaging methods like radiographs, DXA, MRI, and QCT for monitoring bone features in KOA. pQCT captures a cross-sectional "slice" of bone, allowing for the measurement of volumetric bone mineral density and specific compartment measurements of cortical and trabecular bone, along with area measures. Compared to traditional QCT imaging, pQCT delivers a lower radiation dose, making it preferable for repeated measures. pQCT is quick, non-invasive, and has shown reliability in individuals with and without KOA, with high intraclass correlation coefficient (ICC) scores in trabecular regions of interest [23]. Additionally, a previous study of pQCT by Sievänen et al. demonstrated excellent test-retest reliability for trabecular density, cortical density, and cross-sectional area (CSA), however, authors stated that increased soft tissue thickness may impact reliability [24]. Given the increased risk of KOA in individuals with obesity, reliable knee assessment protocols are needed for larger individuals.

The primary objective of this pilot study was to replicate the reliability findings of Sievänen et al. for pQCT measures at the knee, while extending the assessment to additional relevant anatomic locations, including the femoral condyles and medial and lateral regions of the tibia. The secondary objective was to examine whether intra-rater bias and precision of repeated measures differed substantially between adults with and without obesity

## Methods

### Participants and recruitment

The study included adults aged 23 years and older who were capable of walking 100 feet without an assistive device and able to transfer themselves independently into and out of the machine. Those who were pregnant or had prior surgeries on the right knee were excluded. Participants were recruited from the community and previous studies in which individuals agreed to be contacted for future research. Since this pilot study aimed to assess the test-retest reliability, participants were not selected based on any history of knee pathology and were included as a convenience sample. Participants were matched based on body mass index (BMI), specifically, individuals with obesity (BMI ≥ 30 kg/m2) were matched to those without obesity (BMI < 30 kg/m2). Matched individuals could not have a BMI within 5 kg/m2 of each other. Matches were also within ± 5 years of age and the same sex assigned at birth.

### Ethics approval

All procedures were performed in compliance with relevant laws, institutional guidelines, and the Declaration of Helsinki. This study was approved by the Institutional Review Board of the University of Nebraska Medical Center (IRB: 0132-22-FB approved July 5, 2022). Written consent was obtained from participants at the study visit prior to assessment.

### Sample size

The sample size was determined based on ICCs from previous research. Studies reported that pQCT scan outcomes had ICCs ranging from 0.83 to 0.99 at two proximal tibia sites [23]. A sample of 50 participants was determined to be sufficient to detect a correlation between measures of 0.4 and 0.8 vs no correlation with 0.83 and > 0.99 power, respectively using a two-sided test for correlation and a significance level of 0.05.

### pQCT measurement process

A single trained radiographic technician conducted two independent pQCT scans using a Stratec XCT 3000 (Stratec Medizintechnik GmbH, Pforzheim, Germany) during a single visit to the clinical research laboratory. Participants were seated in the scanner, and only the right tibiofemoral joint was scanned to minimize participant burden. The technician obtained a scout image (preliminary scan of the joint) to establish the relative anatomical position to align images. From this, the densest region of the endplate on the medial side of the proximal tibia and distal femur was identified and served as reference points to align scans at each bone. Confirmation of alignment relative to the endplate was conducted to ensure the accuracy of scans between measurements.

The pQCT scan imaged an area 12mm distal to the medial tibial reference point and 2mm proximal to the medial femoral reference point, utilizing a fixed offset measurement. Scan speed was 20mm/s with 0.4mm voxel size and slice thickness was 2.2mm. Tibial scans predominantly captured trabecular bone, while femoral scans primarily captured cortical bone. Between trials, participants exited the pQCT and walked around before the process was repeated.

Images were analyzed using the Stratec XCT Analysis Software (version 6.20). The software was used to draw regions of interest (ROIs) and collect density and structural measurements at each site for the two trials. Bone measures captured included CSA of the total proximal tibia and total, trabecular, and cortical density of the total, medial, and lateral regions of the proximal tibia. Total, trabecular, and cortical density, and CSA of the medial and lateral condyles were separately captured at the distal femur. Strength estimates can be calculated from these measures, so only component measures are presented.

To capture the femoral condyles, a rectangular ROI was manually drawn around the medial and lateral condyles. An irregular ROI was used to exclude fragments outside the condyles if needed. To capture the total, medial, and lateral regions of the tibia, a rectangular ROI spanning the medial to lateral edges was manually drawn to identify the tibial center, after which the tibia was semi-automatically divided into medial and lateral hemispheres.

An irregular ROI was drawn around the outside edges of the tibia on the separate medial and lateral sides, excluding the fibula and/or any fragments. The ROI was minimized to capture the contour of the bone so that the cortical bone created the outside border of the ROI. Lastly, an irregular ROI was drawn to capture the entire tibia. Once the five ROIs were drawn, the 2mm femoral sites and 12mm tibial sites were analyzed with the following parameters: contour mode 31, threshold 169 (threshold 0 for medial and lateral tibial hemispheres), peel mode 2, and a threshold of 500.

**Fig. 1:**
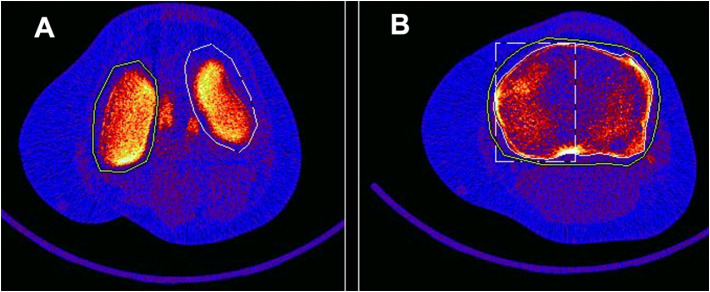
Example of femoral condyle and tibia regions of interest from a peripheral quantitative computed tomography image.

One rater performed and analyzed all scans. Representative ROIs are shown in Fig. 1. **N**

A: Axial plane of distal femoral condyles 2mm from medial femoral endplate reference with regions of interest defined for both the medial and lateral femoral condyles. B: Axial plane of proximal tibia 12mm distal from medial tibial endplate reference with regions of interest defined for the total tibia, and the medial and lateral hemispheres.

The full ROI drawing protocol is provided in **Supplementary File 1.**

### Statistical analysis

Analyses assessed bias and precision of pQCT bone measures using coefficient of variation root mean squared (CV_rms_), reliability coefficient (R), and Bland-Altman analysis. CV_rms_ quantified variability between repeated measures relative to the group mean [25]. The reliability coefficient, defined according to Sievänen et al. (1998): [R=100(1−SD_meas_^2^/SD_biol_^2^) in percentage], was calculated to allow direct comparison with prior work and reflects measurement consistency independent of random error [24]. Bland-Altman analysis evaluated agreement between scans and identified any systematic bias. Reliability analyses were conducted separately for participants with and without obesity to explore whether soft tissue differences might influence measurement bias. Although the pilot study did not statistically compare groups, any observed patterns could inform areas for investigation.

The software package used was R version 4.4.1 (2024-06-14 ucrt) "Race for Your Life" Copyright (C) 2024 The R Foundation for Statistical Computing Platform: x86_64-w64-mingw32/x64. The statistician programmed Bland-Altman analyses and reliability coefficients to match the procedures used by Sievänen et al., allowing direct comparison of findings. This study was reported in compliance with the Guidelines for Reporting Reliability and Agreement Studies (GRRAS) [26].

## Results

The study sample included 52 participants (mean age 50 years [SD: 19.42, range: 23-79], 81% female, BMI 27.98 kg/m2): 26 in the non-obese group (mean age 49.7 years [SD: 20.1, range: 23-74], 81% female, BMI 21.8 kg/m2) and 26 in the obese group (mean age 49.5 years [SD: 19.1, range: 23-79], 81% female, BMI 34.2 kg/m2). All participants completed both scans, but one scan for a single individual was removed due to incorrect scanning procedures.

### Coefficient of variation root mean squared results

Tibial density CV_rms_ were excellent across the total sample and remained consistent within obese and non-obese groups at all regions. CV_rms_ for density measures of the obese group ranged from 1.8% (cortical density, medial tibial hemisphere) to 3.7% (total density, medial tibial hemisphere). Similarly, CV_rms_ density values of the non-obese group ranged from 1.6% (cortical density, medial tibial hemisphere) to 3.6% (cortical density, total tibia). In general, CV_rms_ values were greater for CSA than nearly all density values, regardless of obesity status. CV_rms_ for CSA ranged from 3.8% (total tibia) to 5.2% (medial tibial hemisphere) for the obesity group and 2.8% (total tibia) to 4.6% (lateral tibial hemisphere) for the non-obese group.

At the medial femoral condyle, CV_rms_ density values demonstrated excellent precision across the total sample, with slightly higher values in the obese group compared to the non-obese group, ranging from 0.8% (cortical density, non-obese) to 3.9% (total density, obese). CSA measures for both groups were less precise (11.5%, obese and 8.4%, non-obese).

Precision was lower in all lateral femoral condyle measures compared to other sites. The non-obese group demonstrated greater precision compared to the obese group, with 4.4% vs. 7.9% (cortical density) and 7.5% vs. 17.4% (total density), respectively. CSA measures were not satisfactory for either group, with 21.8% for non-obese and 30.1% for obese. Table 1, Table 2 report CV_rms_ for density and area measures, overall and by obesity group, respectively.

**Table 1:** Total, trabecular, and cortical density and cross-sectional area: Bland-Altman estimated bias, 95% confidence intervals, and agreement limits overall (N = 51).

	Mean (SD)	Range	CV_rms_ (%) (R)	95% limit of agreement	Relative 95% limit of agreement
**Lateral** **Femoral Condyle**^a^*†					
ToD mg/cm^3^*	355.35 (58.08)	187.9-454.1	14.6 (-0.02)	3.92 ± 115.16	1.1% ± 32.41%
TrD mg/cm^3^*	322.56 (38.77)	187.9-372.6	10.8 (0.07)	4.49 ± 73.2	1.39% ± 22.69%
CoD mg/cm^3^†	489.1 (40.38)	408.4-565.8	6.7 (0.24)	1.97 ± 68.83	0.4% ± 14.07%
CSA cm^2^†	555.76 (241.24)	44.48-1166.4	26 (0.6)	-28.24 ± 300.55	-5.08% ± 54.08%
**Medial** **Femoral Condyle**^a^					
ToD mg/cm^3^	348.51 (54.09)	208.1-541.6	3.4 (0.95)	0.77 ± 24.07	0.22% ± 6.91%
TrD mg/cm^3^	312.16 (35.32)	205.1-370.2	3.2 (0.9)	0.67 ± 21.34	0.22% ± 6.84%
CoD mg/cm^3^	511.35 (28.71)	459-638.6	0.9 (0.98)	-0.4 ± 8.82	-0.08% ± 1.72%
CSA cm^2^	827.77 (158.44)	483.52-1278.24	10.3 (0.63)	3.91 ± 189.62	0.47% ± 22.91%
**Total Tibia**^b^					
ToD mg/cm^3^	188.15 (33.63)	120.2-266.5	2.1 (0.99)	0.58 ± 7.66	0.31% ± 4.07%
TrD mg/cm^3^	179.41 (30.93)	115.8-251.8	2.2 (0.98)	0.59 ± 7.52	0.33% ± 4.19%
CoD mg/cm^3^	703.35 (32.3)	639.8-789	2.8 (0.59)	1.99 ± 40.48	0.28% ± 5.76%
CSA cm^2^	3144.91 (503.94)	2274.88-4665.12	3.4 (0.96)	-9.43 ± 204.47	-0.3% ± 6.5%
**Lateral Tibial Hemisphere**^b^					
ToD mg/cm^3^	198.67 (35.48)	127.3-271.1	2.5 (0.98)	-0.36 ± 9.81	-0.18% ± 4.94%
TrD mg/cm^3^	189.69 (32.69)	125-250.6	2.9 (0.97)	-0.17 ± 10.69	-0.09% ± 5.64%
CoD mg/cm^3^	504.3 (26.38)	450.5-574.5	2.7 (0.74)	0.42 ± 26.3	0.08% ± 5.22%
CSA cm^2^	1353.55 (238.98)	857.92-1951.36	4.5 (0.94)	4.74 ± 118.84	0.35% ± 8.78%
**Medial Tibial Hemisphere**^b^					
ToD mg/cm^3^	192.31 (35.13)	115.2-272.6	3 (0.97)	-0.31 ± 11.09	-0.16% ± 5.76%
TrD mg/cm^3^	181.96 (31.96)	114.1-254.7	2.9 (0.97)	-0.47 ± 10.08	-0.26% ± 5.54%
CoD mg/cm^3^	507.31 (24.68)	461.4-572.4	1.7 (0.85)	0.22 ± 18.52	0.04% ± 3.65%
CSA cm^2^	1373 (244.61)	655.36-1971.04	4.4 (0.94)	5.46 ± 118.51	0.4% ± 8.63%

Abbreviations: ToD, total density; TrD, trabecular density; CoD, cortical density; CSA, cross-sectional area

a Femoral condyles scanned 2mm proximal to medial femoral endplate reference point

b Tibia scanned 12mm distal to medial tibial endplate reference point

⁎ Lateral femoral condyle n=35 for ToD and TrD

† Lateral femoral condyle n=46 for CoD and CSA *†Scan location was calibrated based on the medial femoral condyle reference point so not all measures of lateral femoral condyle were captured

**Table 2:** Total, trabecular, and cortical density and cross-sectional area: Bland-Altman estimated bias, 95% confidence intervals, and agreement limits across groups (N = 51).

	Obese n=26*†					Non-Obese n=25*†				
	Mean (SD)	Range	CV_rms_ (%) (R)	95% limit of agreement	Relative 95% limit of agreement	Mean (SD)	Range	CV_rms_ (%) (R)	95% limit of agreement	Relative 95% limit of agreement
**Lateral** **Femoral Condyle**^a^										
ToD mg/cm^3^*	350.17 (61.55)	187.9-454.1	17.4 (-0.55)	8.76 ± 150.24	2.50% ± 42.90%	361.68 (53.72)	244.4-442.2	7.5 (0.75)	-1.83 ± 52.22	-0.51% ± 14.44%
TrD mg/cm^3^*	315.53 (41.75)	187.9-365.3	13.5 (-0.34)	7.73 ± 94.68	2.45% ± 30.01%	331.15 (33.37)	244.4-372.6	5.6 (0.7)	0.64 ± 35.72	0.19% ± 10.79%
CoD mg/cm^3^†	496.09 (39.26)	425.3-565.8	7.9 (-0.25)	4.19 ± 86.12	0.84% ± 17.36%	481.18 (40.61)	408.4-549.6	4.4 (0.73)	-0.68 ± 41.33	-0.14% ± 8.59%
CSA cm^2^†	548.61 (259.5)	44.48-1166.4	30.1 (0.58)	-52.12 ± 330.47	-9.50% ± 60.24%	564.5 (220.2)	112.64-932.64	21.8 (0.64)	0.12 ± 260.06	0.02% ± 46.07%
**Medial** **Femoral Condyle**^a^										
ToD mg/cm^3^	359.34 (56.07)	208.1-541.6	3.9 (0.93)	0.01 ± 29.37	0.00% ± 8.17%	337.25 (50.05)	237.1-434	2.7 (0.97)	1.56 ± 17.44	0.46% ± 5.17%
TrD mg/cm^3^	316.97 (33.92)	205.1-370.2	3.5 (0.86)	-0.77 ± 25.22	-0.24% ± 7.96%	307.16 (36.4)	223.8-367.1	2.6 (0.95)	2.18 ± 16.38	0.71% ± 5.33%
CoD mg/cm^3^	520.35 (30.62)	466.4-638.6	0.9 (0.98)	0.8 ± 9.06	0.15% ± 1.74%	502 (23.41)	459-545.7	0.8 (0.97)	-1.65 ± 8.02	-0.33% ± 1.60%
CSA cm^2^	873.74 (154.48)	604.32-1278.24	11.5 (0.42)	17.48 ± 230.58	2.00% ± 26.39%	779.96 (149.43)	483.52-1091.36	8.4 (0.79)	-10.21 ± 134.01	1.31% ± 17.18%
**Total Tibia**^b^										
ToD mg/cm^3^	194.27 (30.16)	120.2-266.5	2.3 (0.98)	0.34 ± 8.55	0.18% ± 4.40%	181.78 (36.09)	127.6-241.6	1.9 (0.99)	0.84 ± 6.74	0.46% ± 3.71%
TrD mg/cm^3^	183.5 (27.14)	115.8-251.8	2.3 (0.98)	0.38 ± 8.15	0.21% ± 4.44%	175.16 (34.2)	121.3-234.3	2.1 (0.99)	0.81 ± 6.95	0.46% ± 3.97%
CoD mg/cm^3^	709.69 (30.37)	644.5-776.1	2 (0.73)	0.82 ± 30.93	0.12% ± 4.36%	696.75 (33.21)	639.8-789	3.6 (0.43)	3.22 ± 49.05	0.46% ± 7.04%
CSA cm^2^	3273.63 (484.7)	2328.32-4665.12	3.8 (0.94)	-27.18 ± 236.77	-0.83% ± 7.23%	3011.04 (493.02)	2274.88-4470.72	2.8 (0.97)	9.03 ± 161.34	0.30% ± 5.36%
**Lateral Tibial Hemisphere**^b^										
ToD mg/cm^3^	210.23 (29.49)	149.4-271.1	2.7 (0.97)	-0.76 ± 10.79	-0.36% ± 5.13%	186.65 (37.42)	127.3-245.2	2.4 (0.99)	0.06 ± 8.81	0.03% ± 4.72%
TrD mg/cm^3^	198.18 (25.7)	141.1-250.6	3.1 (0.94)	-0.61 ± 11.98	-0.31% ± 6.05%	180.85 (36.86)	125-237.4	2.7 (0.98)	0.28 ± 9.32	0.15% ± 5.15%
CoD mg/cm^3^	510.84 (24.98)	450.5-574.5	1.9 (0.86)	1.64 ± 18.23	0.32% ± 3.57%	497.5 (26.31)	459.3-571.9	3.4 (0.59)	-0.84 ± 32.9	-0.17% ± 6.61%
CSA cm^2^	1387.79 (240.33)	1030.72-1951.36	4.5 (0.93)	-2.79 ± 120.58	0.20% ± 8.69%	1317.94 (234.66)	857.92-1855.04	4.6 (0.93)	12.58 ± 117.46	0.95% ± 8.91%
**Medial Tibial Hemisphere**^b^										
ToD mg/cm^3^	195.41 (30.12)	133.3-272.6	3.7 (0.94)	-0.33 ± 14.12	-0.17% ± 7.23%	189.08 (39.73)	115.2-258.6	1.8 (0.99)	-0.29 ± 6.94	-0.15% ± 3.67%
TrD mg/cm^3^	183.75 (27.66)	126.4-254.7	3.4 (0.95)	-0.26 ± 12.53	-0.14% ± 6.82%	180.09 (36.09)	114.1-247	1.9 (0.99)	-0.68 ± 6.9	-0.38% ± 3.83%
CoD mg/cm^3^	514.54 (27.12)	461.4-572.4	1.8 (0.87)	-1.12 ± 19	-0.22% ± 3.69%	499.79 (19.41)	466.6-549.5	1.6 (0.78)	1.61 ± 17.99	0.32% ± 3.60%
CSA cm^2^	1412.3 (248.36)	1018.24-1971.04	5.2 (0.92)	8.01 ± 141.2	0.57% ± 10.00%	1332.13 (236.2)	655.36-1791.2	3.6 (0.96)	2.8 ± 91.85	0.21% ± 6.89%

Abbreviations: ToD: total density; TrD: trabecular density; CoD: cortical density; CSA: cross-sectional area

a Femoral condyles scanned 2mm proximal to medial femoral endplate reference point

b Tibia scanned 12mm distal to medial tibial endplate reference point

⁎ Lateral femoral condyle obese n=19 and non-obese n=16 for ToD and TrD

† Lateral femoral condyle obese n=25 and non-obese n=21 for CoD and CSA *† pQCT scan area was calibrated based on the medial femoral condyle reference point so not all densities of lateral femoral condyle were captured

### Reliability coefficient results

R values demonstrated excellent precision for total and trabecular density, along with CSA across all areas of the tibia for the total sample and within both obese and non-obese groups, ranging from 0.94-0.99 for total and trabecular density and 0.92-0.97 for CSA. Although slightly lower, cortical density of the tibia had higher R values in the obese group ranging from 0.73-0.87, while the non-obese group ranged from 0.43-0.78.

The medial femoral condyle exhibited strong precision in both groups for all densities with R values ranging from 0.86-0.98 and 0.95-0.97 in the obese and non-obese groups, respectively. CSA R values were lower, with values of 0.42 and 0.79, respectively.

Measurements from the lateral femoral condyle demonstrated lower precision across both groups. In the non-obese group, R values for total, trabecular, and cortical density were 0.75, 0.70, and 0.73, respectively. In contrast, the obese group was unreliable, with R values of -0.55, -0.34, and -0.25 for the same measures. CSA R values were low overall, with an R value of 0.58 in the obese group and 0.64 in the non-obese group. Table 1, Table 2 report R coefficient values for density and area measures, overall and by obesity group, respectively.

### Bland-Altman analysis results

Bland-Altman analysis of pQCT density measures demonstrated no bias and excellent agreement for all density and area measures across tibial sites, with no substantial differences between obesity groups. Relative bias values for density values ranged from -0.36% (total density, lateral tibial hemisphere) to 0.32% (cortical density, lateral tibial hemisphere) in the obese group and -0.38% (trabecular density, medial tibial hemisphere) to 0.46% (total, trabecular, and cortical density, total tibia) in the non-obese group. CSA relative biases values were slightly greater, ranging from -0.83% (total tibia) to 0.57% (medial tibial hemisphere) in the obese group and 0.21% (medial tibial hemisphere) to 0.95% (lateral tibial hemisphere) in the non-obese group. Biases were consistently centered around 0 with no apparent effect due to obesity status.

However, obesity was associated with slightly wider 95% limits of agreement at nearly all tibia density and area measures, resulting in a small reduction in precision. In the obese group, 95% limits of agreement ranged from ± 3.57% (cortical density, lateral tibial hemisphere) to ± 10.00% (CSA, medial tibial hemisphere). In the non-obese group, they ranged from ± 3.60% (cortical density, medial tibial hemisphere) to ± 8.91% (CSA, lateral tibial hemisphere).

Analysis of the medial femoral condyle demonstrated no significant bias across all density measures, with the narrowest limits of agreement in analyzing cortical density and no substantial differences by obesity status (± 1.74% for obese and ± 1.60% for non-obese). Relative bias values ranged from -0.24% (trabecular density) to 0.15% (cortical density) for the obese group and -0.33% (cortical density) to 0.71% (trabecular density) for the non-obese group. CSA measures were not satisfactory overall, with relative biases values (± 95% limits of agreement) of 2.00% (± 26.39%) in the obese group and 1.31% (± 17.18%) in the non-obese group.

All measures of the lateral femoral condyle exhibited bias and poor agreement in the obese group, with relative bias values ranging from -9.50% (CSA) to 2.5% (total density) and 95% limits of agreement ranging from ± 17.36% (cortical density) to ± 60.24% (CSA). In contrast, the non-obese group showed no bias at this site, with relative bias values ranging from -0.51% (total density) to 0.19% (trabecular density). However, the 95% limits of agreement were still wide, ranging from ± 8.59 (cortical density) to ± 46.07% (CSA).

**Fig. 2:**
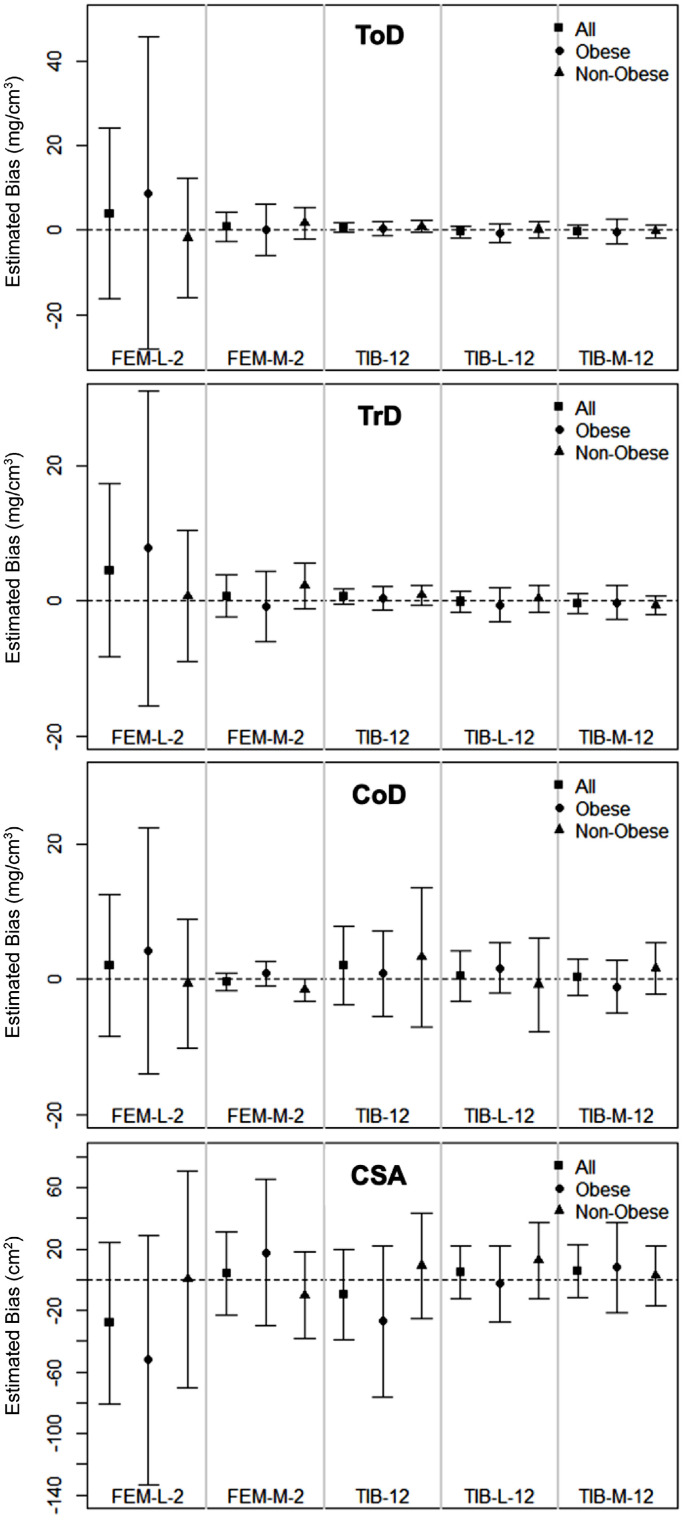
Total, trabecular, and cortical density and cross-sectional area summary of Bland-Altman biases across groups.

Detailed bias values for all pQCT measures, along with corresponding 95% limits of agreement, are provided in Table 1, Table 2 for the overall sample and by obesity group, respectively. Fig. 2 presents a summary of the Bland-Altman plots for visual reference, with full Bland-Altman plots provided in **Supplementary File 2**.

Representative Bland-Altman plot summary with estimated bias and 95% limits of agreement comparing total density, trabecular density, cortical density, and cross-sectional area between the total, obese, and non-obese groups at the lateral femoral condyle, medial femoral condyle, total tibia, lateral tibial hemisphere, and medial tibial hemisphere. Abbreviations: ToD: total density; TrD: trabecular density; CoD: cortical density; CSA: cross-sectional area; FEM-L-2: lateral femoral condyle 2mm; FEM-M-2: medial femoral condyle 2mm; TIB-12: total tibia 12mm; TIB-L-12: lateral tibial hemisphere 12mm; TIB-M-12: medial tibial hemisphere 12mm

## Discussion

This pilot study assessing the intra-rater reliability of pQCT in evaluating bone of the knee joint found that pQCT demonstrates no bias and excellent precision in measuring total, trabecular, and cortical density at all areas of the tibia and the medial femoral condyle, with no substantial differences due to obesity status. pQCT showed no systematic biases in CSA measures at the tibia, however, 95% limits of agreement were wide, impacting precision of the measure. CSA measures of the medial femoral condyle were unreliable. All measures of the lateral femoral condyle were unreliable due to inconsistent capture since the pQCT was aligned based on the medial femoral condyle.

Our findings for trabecular and cortical density of the tibia align with Sievänen et al., who previously demonstrated the reliability of pQCT measurements of the proximal tibia and distal femur [24]. However, they did not assess the medial and lateral hemispheres of the tibia, and their ROI of the distal femur (5% of the approximated limb length) did not capture the condyles. These differences limit the ability to compare our results with theirs at these sites.

Sievänen et al. reported that increased soft tissue thickness affected reliability in an in vitro model but did not examine this relationship in human subjects [24]. Long bone ends in joint regions typically have thin layers of soft tissue, so they noted the impact of increased tissue thickness at these sites may have a minimal impact on reliability. Our study extended the work of Sievänen et al. by addressing this gap, comparing pQCT reliability at the knee between sex- and age-matched (±5 years) participants with and without obesity. In general, presence of obesity did not result in substantial bias differences, however, it did typically result in wider ranges of 95% limits of agreement, slightly impacting the precision of results when compared to non-obese individuals. Despite this small reduction in precision, imaging knees of persons with obesity with pQCT was reliable.

Bland-Altman bias values and 95% limits of agreement for total and trabecular density of the tibia reported in this study are minimal. Overall, estimated biases for total and trabecular density of the tibia were consistently less than 1 mg/cm^3^. Although the obese group showed slightly wider 95% limits of agreement in total and trabecular density measures compared to the non-obese group, the limits of agreements never varied by more than ± 1.5 mg/cm^3^. This difference is not clinically relevant as Bennell et al. found significant density differences of 20-30 mg/cm^3^ in specific areas of trabecular bone of the tibia when comparing individuals with moderate KOA to those without KOA [23], indicating the small agreement differences should not impede the ability of pQCT to detect minor early bone changes of KOA. Measures of cortical density demonstrated slightly better precision in the obese group compared to the non-obese group. This is likely due to increased density and thickness of cortical bone in those with obesity, with a mean density of 709.69 mg/cm^3^ compared to 696.75 m/cm^3^ in those without obesity.

The lower R values of cortical density at the tibia likely result from the narrow range of mean densities among participants, where small measurement differences reduce R values. The true differences between scans were small as demonstrated by the low CV_rms_ values indicating reliability of tibial cortical density measures.

The selected ROI at the distal aspect of the femoral condyles likely contributed to the imprecision of some measures due to the thin cortical bone that rapidly transitions to trabecular bone, causing significant variations with small changes in positioning. An ROI located at a more proximal region of the femoral condyles would likely provide better reliability due to the more uniform shape and structure of bone at that region. Additionally, the medial femoral condyle had greater reliability as the scans were aligned relative to the medial endplate. Using separate reference lines to capture each condyle individually could improve the performance of pQCT at the lateral condyle.

Our findings support the use of pQCT for assessing tibial bone changes in KOA, demonstrating reliable measurements even in high-risk individuals with obesity. Although early KOA changes after injury primarily affect trabecular bone, our results indicate that pQCT can reliably assess various bone measures across the entire tibia and medial femoral condyle [6,23,[27], [28], [29]]. Accurate assessment of bone density and structure at various areas provides valuable insights into the role of bone changes in the onset and progression of KOA. Since similar KOA changes occur in both the tibia and femur [30], future protocols should capture the femoral condyles at a more proximal region and incorporate two reference points for improved reliability. pQCT is a reliable, quick and non-invasive method to assess tibial and medial femoral bone density with a low radiation dose. Our pQCT scanning protocol exposed participants to ∼200x less radiation than a comparable knee QCT scan [18], while also offering a three-dimensional (volumetric) assessment, providing a more accurate measurement of true bone density. High-resolution peripheral CT scanners are available, however, their practical application is currently limited by their expense and small gantry size, which makes them unable to accommodate large limb sizes or assess the proximal tibia [31]. In addition to the advantages of pQCT imaging, our fixed offset measurement does not require the use of physical palpation, which often causes positioning issues when imaging patients with obesity. By using the scout image, radiographic technicians can calibrate the machine to start from the same reference point each time and image proximally or distally from that reference point. This method significantly reduces the challenges of positioning and identifying landmarks often encountered when imaging individuals with obesity, resulting in enhanced precision of scans.

## Strengths and limitations

Our study is limited by the alignment reference point used to measure the femoral condyles. The scan was aligned on the medial femoral condyle endplate to measure an area 2mm proximal to the reference line. This approach resulted in inconsistent capture of the lateral femoral condyle as small changes in limb position caused greater positional variation of the lateral femoral condyle. Thus, this approach found bias and low precision at this site. To address this issue, separate scans could be captured for each condyle, with alignment based on each condyle’s reference point.

A minor limitation of the study is the voxel resolution of 0.4mm. The transition of the cortical to trabecular bone is thin at our ROI of the femoral condyles, so the voxel may overlap soft tissue, cortical bone, and trabecular bone, producing partial volume effects that reduces reliability at this site. The voxel resolution could have been improved, however, that would increase scan time and potential for movement artifacts. An additional limitation is that predetermined acceptable limits of agreement were not established prior to conducting the study. However, pQCT is not used for clinical application so reference values are not available.

The majority of our participants were female (81%), reducing the generalizability of our study results.

Strengths of this study include matching participants with and without obesity by age (± 5 years) and sex, along with a broad age range (23 to 79 years old). Although participants between 30 to 40 years old were underrepresented, this range allowed assessment across a wide spectrum of bone densities. A key strength of this study is the evaluation of pQCT reliability in individuals with obesity, addressing known challenges related to increased soft tissue thickness and image acquisition in a high-risk KOA population.

## Conclusion

This study demonstrates that pQCT provides reliable measurements of bone density at the tibia and medial femoral condyle, showing no substantial differences in reliability between individuals with and without obesity. pQCT shows promise as a method for early detection of KOA, offering potential insights into its pathogenesis and serving as a valuable tool to monitor bone changes in high-risk individuals. Its decreased precision in evaluating CSA and alignment-related issues of femoral scans suggests areas for improvement and further investigation. Potential solutions to address these issues include using a foot-holding device to maintain consistent leg positioning which may improve CSA precision, and capturing a second image aligned with a more proximal region of lateral femoral condyle to address the alignment issues of femoral scans. Future trials can utilize pQCT to investigate the specific areas and temporal sequence of bone changes that occur during the development of KOA.

## Author contributions

Reece Blay designed the experiment, collected data, interpreted data, created the tables and figures, and drafted the manuscript

Christopher Wichman performed data analysis and visualization of data

Yvonne M. Golightly drafted the manuscript

Laura Bilek assisted with experimental design and drafted the manuscript

## Role of funding source

This research did not receive any specific grant from funding agencies in the public, commercial, or not-for-profit sectors.

## Declaration of competing interest

The authors declare that they have no known competing financial interests or personal relationships that could have appeared to influence the work reported in this paper.

## Appendix Supplementary materials

Image, application 1

Image, application 2
