# Supplementary figures and images for: Assessing intra-rater reliability of peripheral quantitative computed tomography in knee joint bone evaluation on individuals with and without obesity: A GRRAS study
Source: Osteoarthr Imaging. 2026 Mar 7;6(1):100393. doi: 10.1016/j.ostima.2026.100393 (PMC13228721; doi:10.1016/j.ostima.2026.100393)

**Supplementary Figure 2: Full Bland-Altman Plots for Tibial and Femoral pQCT Measures**


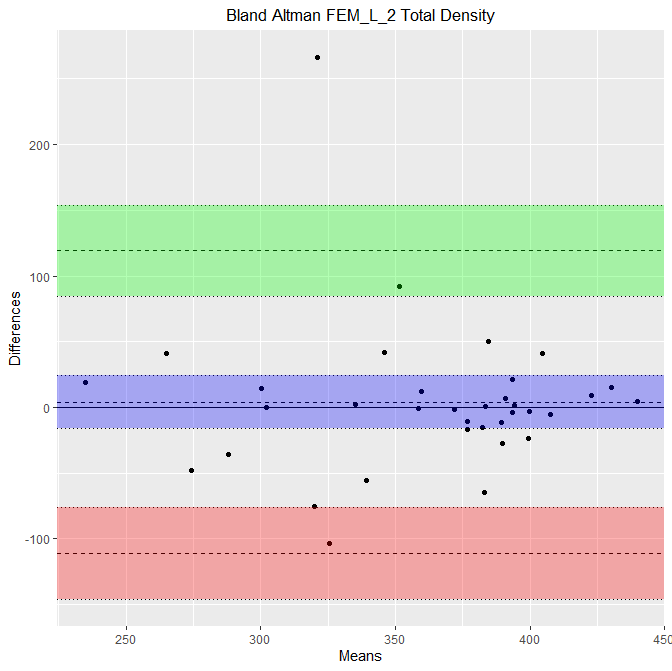


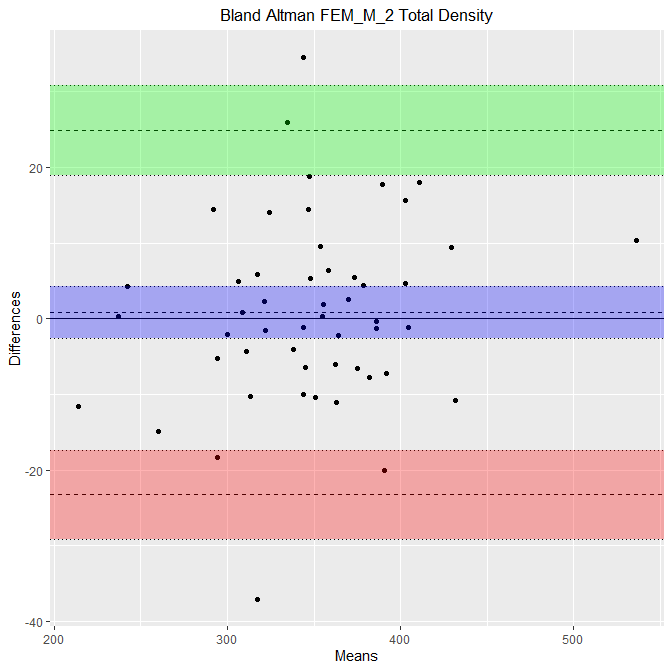


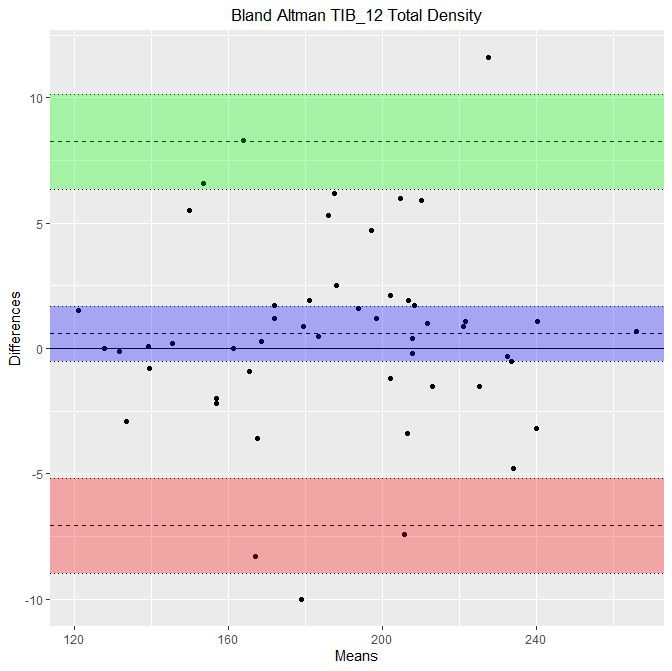


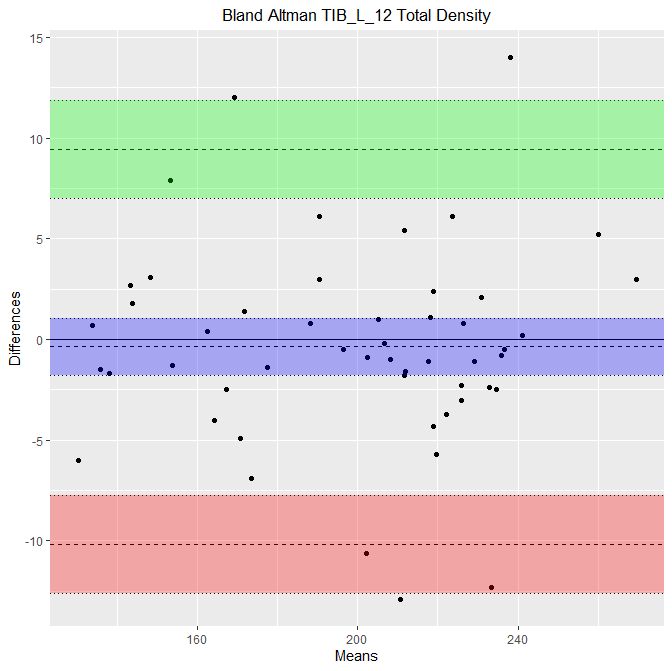


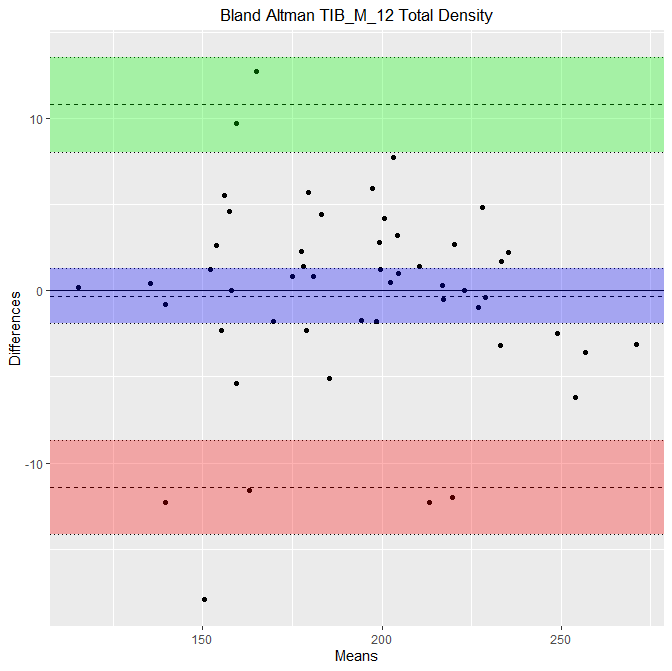


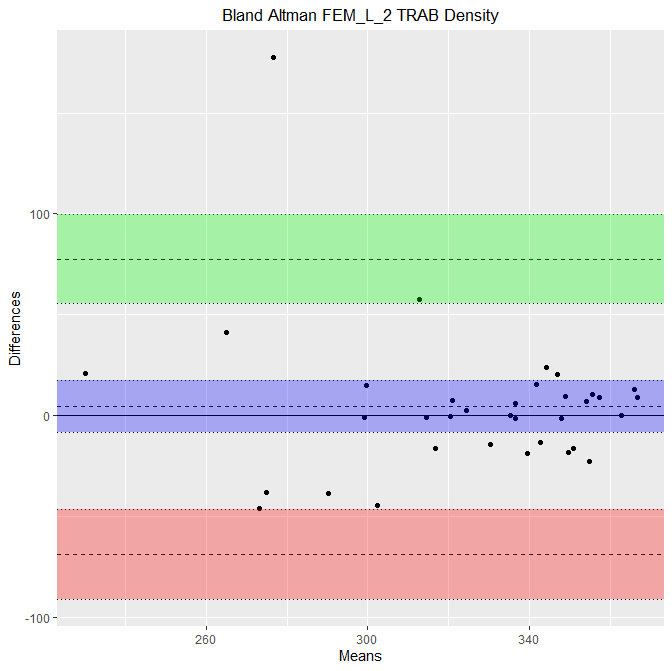


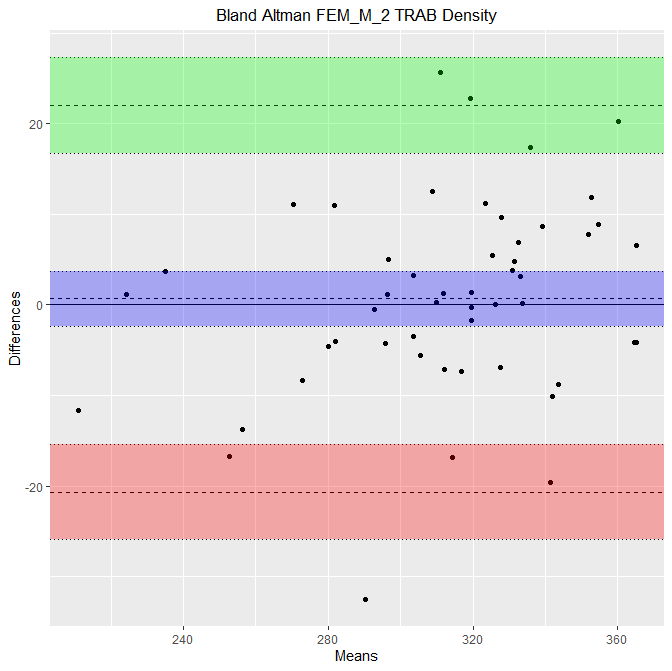


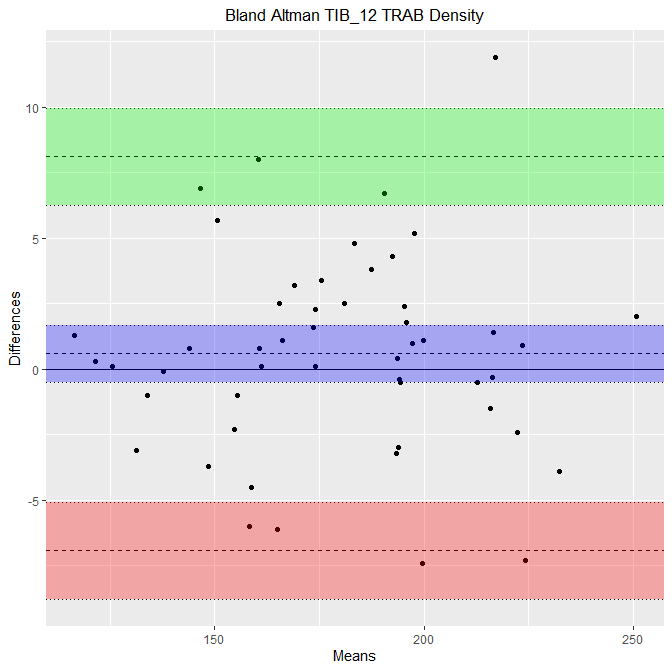


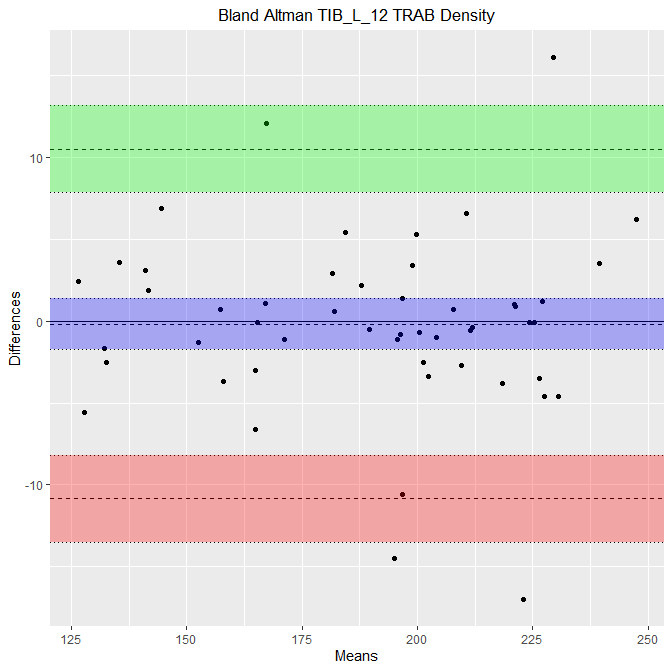


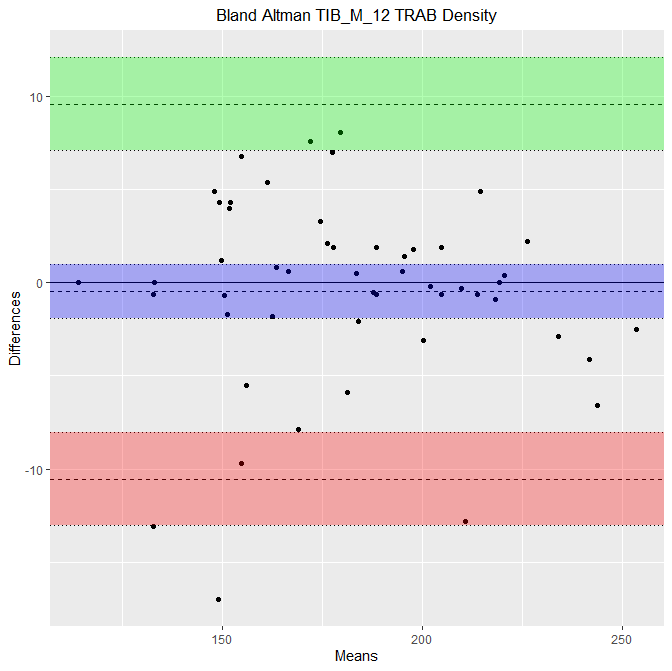


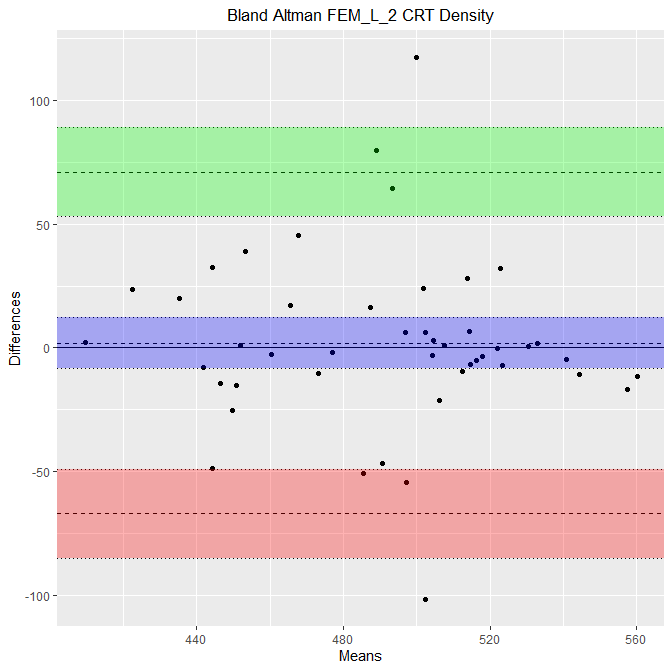


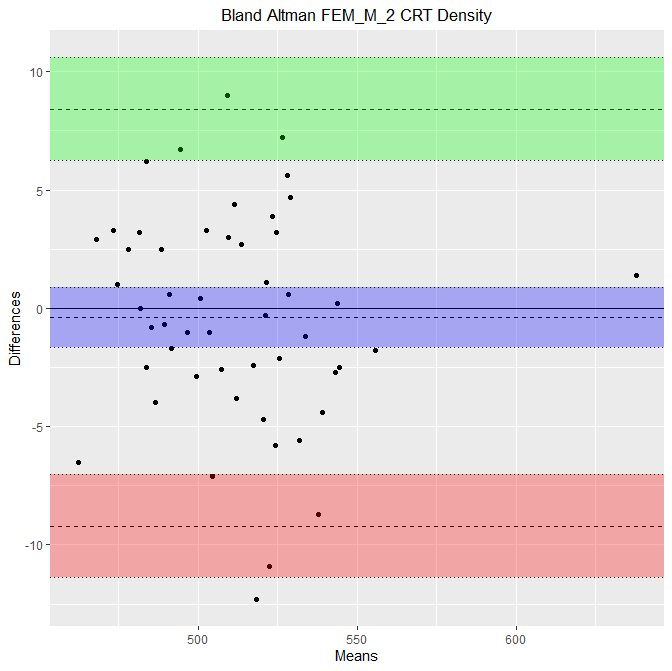


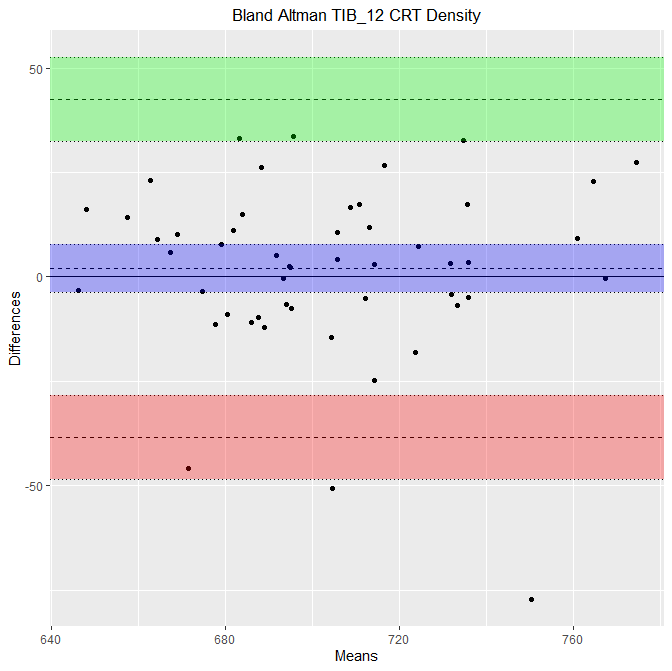


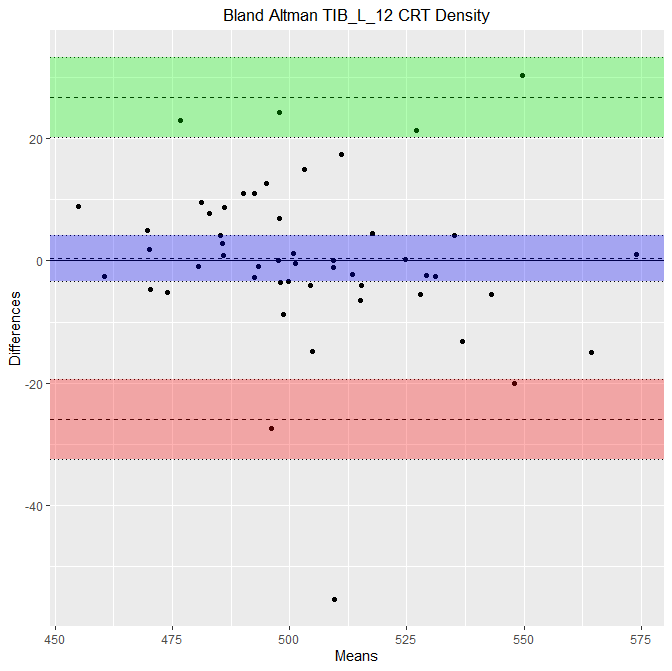


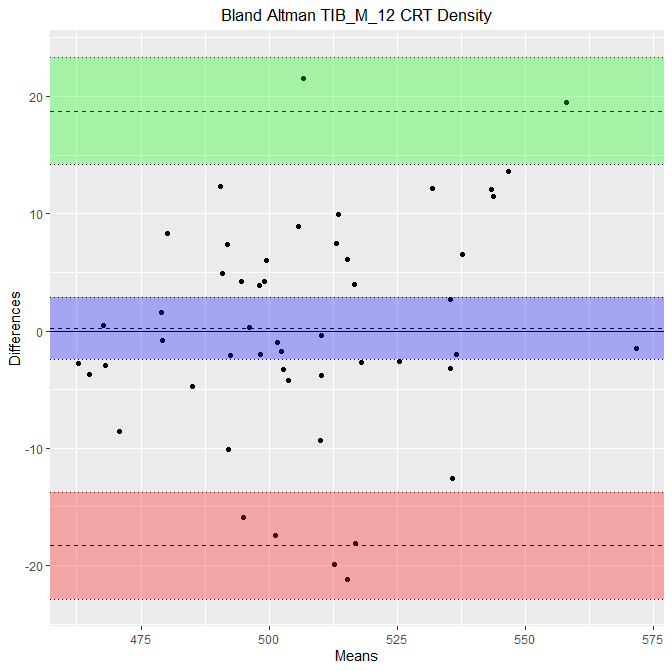

Supplement: Supplementary file 1 [file mmc1.docx]
