# Supplementary material for: Assessing intra-rater reliability of peripheral quantitative computed tomography in knee joint bone evaluation on individuals with and without obesity: A GRRAS study
Source: Osteoarthr Imaging. 2026 Mar 7;6(1):100393. doi: 10.1016/j.ostima.2026.100393 (PMC13228721; doi:10.1016/j.ostima.2026.100393)
Supplement: Supplementary file 2 [file mmc2.docx]

**Supplementary File 1: Manual ROI Drawing Protocol for Tibial and Femoral pQCT Scans**


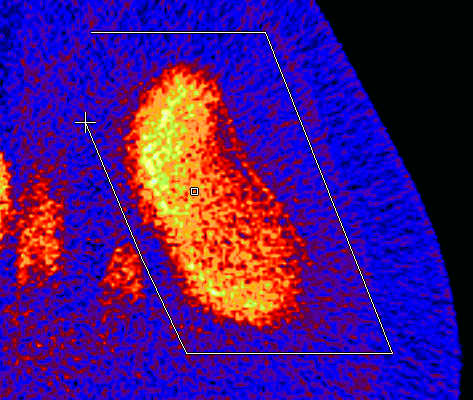


Exclude bone

Horizontal first line

ROI Start

ROI End

- Always start the irregular ROI above and to the left of the condyle
- Keep the first segment horizontal
- Do not try to “contour” the bone, and keep a distance away as shown
- Exclude any bone like objects, small points are Ok


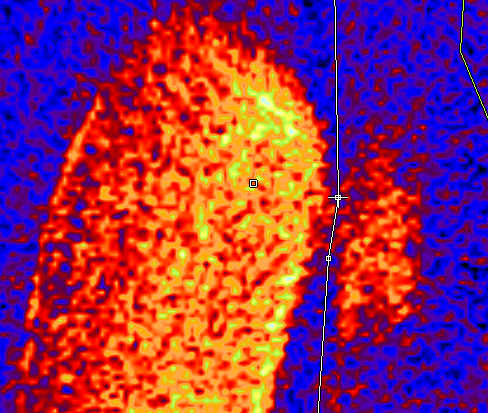


Position points centered between the two bones

- Use “Z” or “shift Z” to zoom in on details
- Try to dissect boney objects through the mid-point


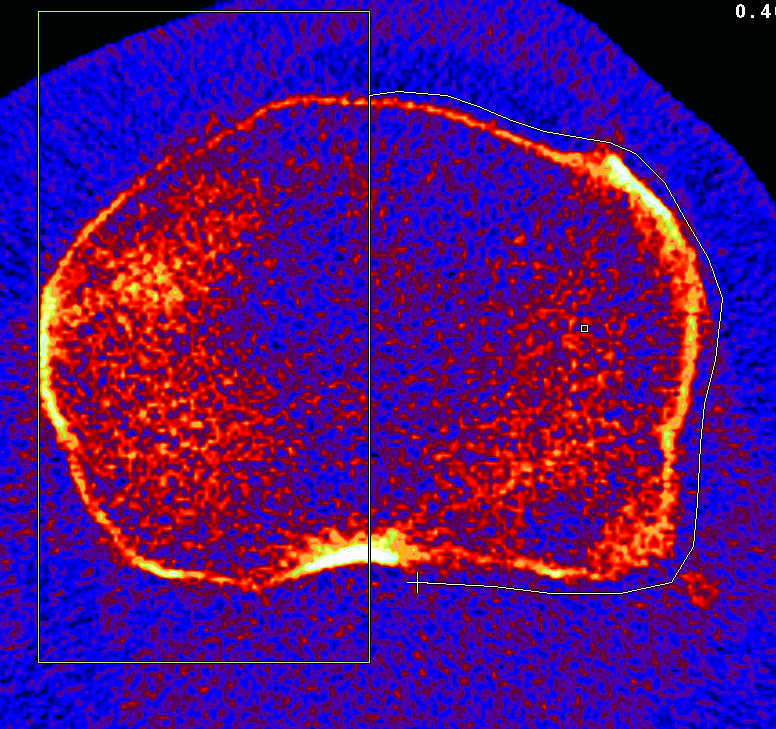


ROI Start when drawing on the right half

On the tibia follow the contour of the bone as shown here

ROI End

- As you draw along the contour of the bone stay close and try to exclude any dense points outside the bone


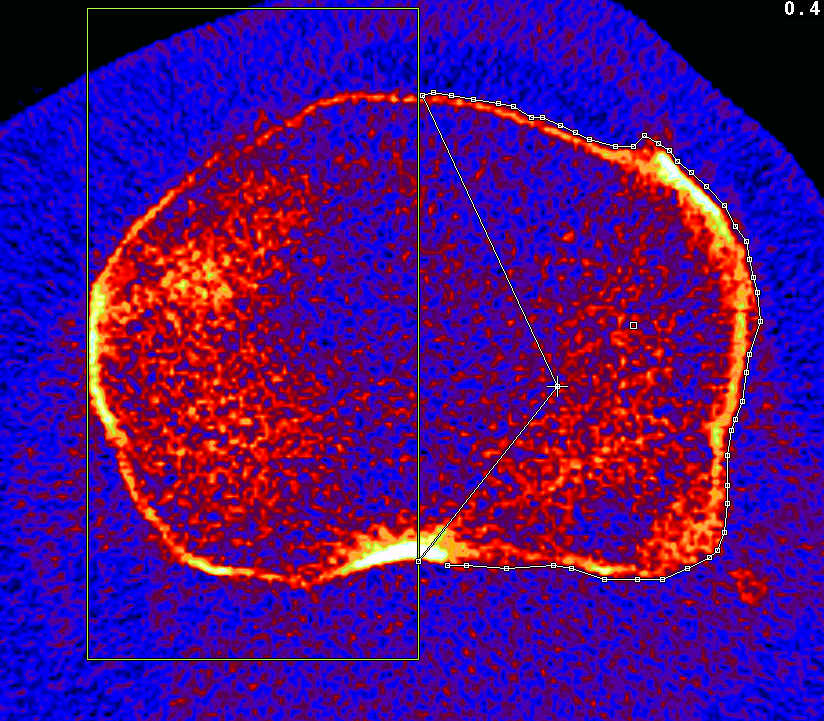


Adjust points to center

Hover over this point with your mouse and delete it.

- Pressing “M” will minimize the points to the contour of the bone.
- Only adjust points if they are obviously incorrect!
- Delete any points that pop up on the center line
- Adjust the points so they are on the center


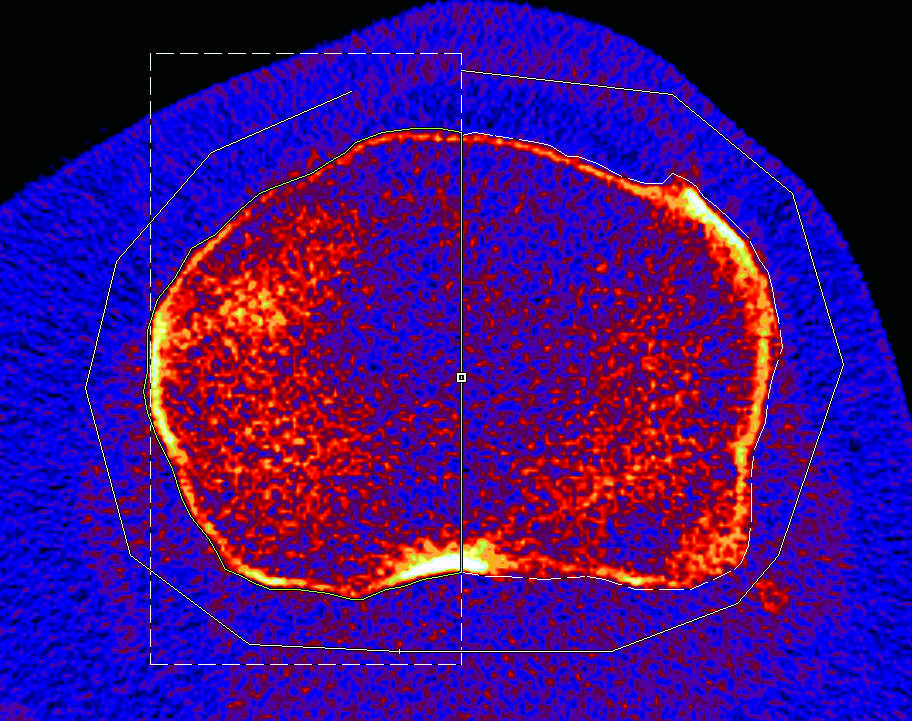


Exclude any larger densities like the fibula

Start ROI on the center line

- Start the TIB_12 ROI on the center line above the tibia
- Exclude the fibula
- Keep a good distance as shown
